# Skin flap shift is associated with postoperative complications after cranioplasty: a retrospective cohort study

**DOI:** 10.3389/fneur.2025.1714893

**Published:** 2025-12-11

**Authors:** Shangshuo Liu, Ronglun Dang, Yida Li, Liyuan Ma, Wenke Zhou

**Affiliations:** 1 Department of Neurosurgery, The First Affiliated Hospital of Henan Medical University, Xinxiang, Henan, China; 2 Center for Surgical Oncology, Xinjiang Medical University Affiliated Cancer Hospital, Xinjiang, China

**Keywords:** skin flap shift, cranioplasty, post-operative complications, predictive factors, cohort study

## Abstract

**Background:**

The incidence of postoperative complications following cranioplasty (CP) procedures remains relatively high, which has a significant impact on patient prognosis. While current research on predictive factors for complications has focused primarily on patient demographics, the timing of surgery and material selection, the association between skin flap shift and complications has yet to be systematically evaluated.

**Objective:**

To investigate the correlation between skin flap shift and postoperative complications following CP.

**Methods:**

A cohort of patients undergoing CP was enrolled and categorized into postoperative complication and no-complication groups. First, we conducted a univariate analysis on the following variables: age; gender; medical history; and surgical variables. Variables with a *p*-value of ≤0.2 in the univariate analysis were included in the multivariate logistic regression analysis. For the continuous variables, ROC curves were used to determine the optimal cut-off values for predicting complications. These values were then converted into binary variables for the multivariate analysis.

**Results:**

Univariate analysis demonstrated that the differences in the materials utilized for repair, intraoperative blood loss, and skin flap shift between the two groups were statistically significant. The optimal cutoff values for intraoperative blood loss and skin flap shift, as determined by ROC curve analysis, were identified as 175 mL and 13.55 mm, respectively. Multivariate logistic regression analysis identified skin flap shift to be independently associated with postoperative complications after CP. (OR: 3.239, 95% CI: [1.450–7.237], *p* = 0.004). The area under the curve for predicting postoperative complications based on skin flap shift was 0.719 (95%CI: 0.646–0.797).

**Conclusion:**

Skin flap shift was independently associated with postoperative complications following CP surgery. Patients with flap displacements exceeding 13.55 mm are at an increased risk of experiencing such complications.

## 1 Introduction

Craniectomy decompression is currently one of the primary neurosurgical interventions for the alleviation of elevated intracranial pressure and the saving of patients’ lives (1). This procedure, involving the excision of a cranial bone flap and craniotomy, is employed in patients with severe traumatic brain injury and significant cerebral oedema or swelling (2). The treatment is effective in reducing intracranial pressure and preventing further neurological damage, with a reported high success rate (3–5). However, it must be noted that the treatment inevitably results in a cranial defect. Cranioplasty (CP) is defined as the surgical procedure of filling and repairing the defect left after decompressive craniectomy using various reconstructive materials. It is currently one of the most routine procedures in neurosurgery. A growing body of evidence indicates that cranial reconstruction not only restores the morphology of the cranial cavity, achieving aesthetic restoration, but also plays a significant role in the recovery of the patient’s neurological function (6).

Although the surgical technique for cranioplasty is well-established and the optimal timing for repair is widely accepted, postoperative complications can vary significantly. These include superficial or deep surgical site infections, poor wound healing, tissue non-union, seroma or hematoma formation beneath the galea aponeurotica or bone flap, cranial bone resorption, exposure of the graft material, various delayed haematomas, seromas, cerebral oedema or cerebral infarction, and new-onset epilepsy (7). Severe complications necessitate reoperation or removal of the bone flap followed by re-repair, while systemic complications may lead to poor patient prognosis or even death (8).

Recent studies on predictive factors for cranial repair complications have predominantly focused on patient demographics, comorbidities, timing of surgery, defect size, implant material selection, and intraoperative techniques (9). Nevertheless, systematic research on the relationship between skin flap shift and postoperative complications remains limited. Research has indicated that bone window depression is an independent risk factor for complications arising in the context of cranial repair (10). Abnormal flap displacement has been demonstrated to be closely associated with postoperative dead space formation, local haemodynamic impairment, and altered biomechanical conditions (11). This has been shown to become a significant precipitating factor for complications such as subcutaneous fluid accumulation and infection. The present study utilized a cohort of 148 CP patients to investigate the relationship between skin flap shift and postoperative complications following CP (12).

## 2 Materials and methods

### 2.1 Sample sources

**Figure 1:**
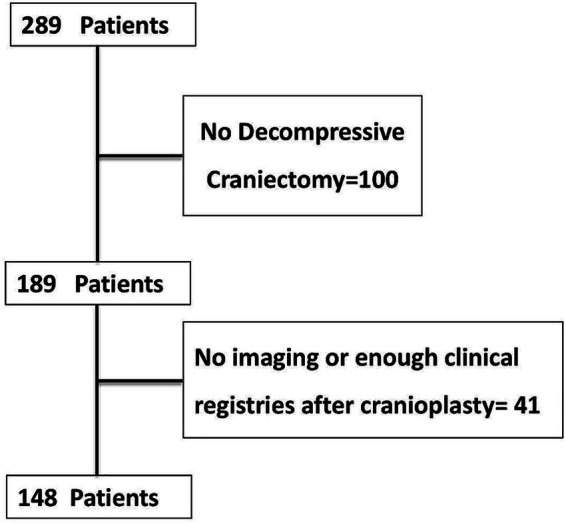
Diagram of the selected patients with cranioplasty from July 2018 and June 2025 included for analysis.

Patients treated at the Department of Neurosurgery at the First Affiliated Hospital of Henan Medical University between July 2018 and June 2025 were selected for this study. Patients who met the following inclusion criteria were deemed eligible for participation: (i) patients who underwent elective titanium mesh CP after first receiving decompressive craniectomy; (ii) possession of comprehensive preoperative thin-slice cranial CT three-dimensional reconstruction imaging data, enabling precise measurement of skin flap shift; (iii) complete clinical documentation; (iiii) signed informed consent. Exclusion criteria are as follows: (i) presence of systemic diseases severely impairing healing (immunodeficiency disorders, long-term immunosuppressant or glucocorticoid use, connective tissue diseases); (ii) pre-existing neurological deficits or infection; (iii) active infection in the surgical area, excessive flap tension, or significant vascular compromise prior to surgery; (iiii) refusal of follow-up by the patient or their family. Based on these criteria, 148 patients were ultimately included: The cohort consisted of 110 males and 38 females, with a median age of 52 years (interquartile range [IQR], 42–58) (Figure 1).

### 2.2 Variables

The medical records system collects patient age, gender, medical history (diabetes, hypertension) and the clinical context for craniectomy (trauma, stroke). Surgical records were used to retrieve data on craniectomy characteristics (choice of CP material, size of cranial defect) and the presence and reasons for reintervention. The duration of surgery was determined based on hospital surgical records. The cranioplasty interval was defined as the difference between the cranioplasty date and the date of decompressive craniectomy.

In our study, all patients underwent a standardized three-month postoperative clinical follow-up. This is consistent with commonly used short-term outcome windows in cranioplasty research, where early complications such as infection, hematoma, subgaleal effusion, wound breakdown, and hydrocephalus typically occur within the first 1–3 months after surgery (7). Complications that occurred within this predefined three-month period were recorded and included in the analysis. The complication group was defined by the development of any complication during follow-up, whereas the no-complication group included patients who remained free of all complications.

**Figure 2:**
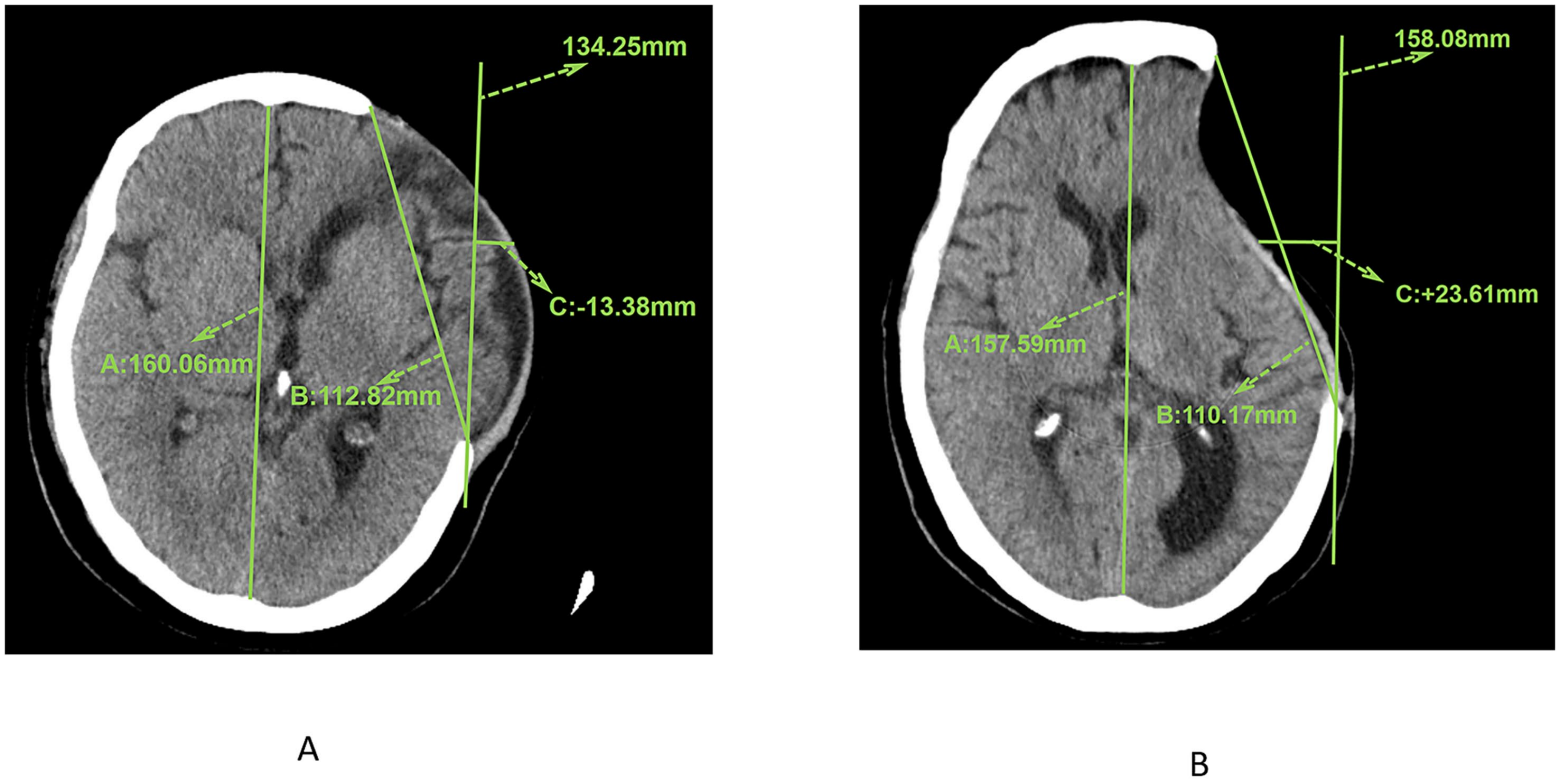
**(A)** CT image showing the measurement results of the maximum axial craniectomy size (110.17 mm) and skin flap shift (23.61 mm). **(B)** CT image showing the measurement results of the maximum axial craniectomy size (112.82 mm) and skin flap shift (13.38 mm).

The measurement of skin flap shift was conducted at the image plane that exhibited the most pronounced deviation. All images employed for the measurement of skin flap shift were obtained 7 days prior to surgery and measured by two associate chief physicians, with excellent inter-observer reliability indicated by an intraclass correlation coefficient (ICC) of 0.923 (95% CI: 0.894–0.945, *p* < 0.001). Initially, the bony platform at the cranial defect site that was furthest horizontally from the midline was selected. A straight line parallel to the midline reference line was drawn from the centre of the defect margin. The skin flap shift was determined by measuring the distance between this line and the flap position at the midpoint of the craniectomy axis. In instances where the flap measurement point did not reach this vertical line (Figure 2A, +23.61 mm), it was deemed positive. Conversely, if the flap measurement point lay lateral to the reference line (Figure 2B, −13.38 mm), it was negative. Cerebral displacement was the underlying factor that resulted in the non-utilisation of the contralateral side as a reference benchmark.

### 2.3 Statistical analysis

Statistical analyses were conducted using SPSS version 26 (IBM Corp, Chicago, USA). Continuous variables were expressed as mean ± standard deviation and analyzed with Student’s t-test, while categorical data were summarized as frequencies and compared with the chi-square test. The normality of data distribution was assessed with the Kolmogorov–Smirnov test. For non-normally distributed continuous variables or ordinal data, the rank-sum test was applied. Single factor analysis with *p* ≤ 0.2 was included in the multi-factor analysis. In the context of continuous variables, the Receiver Operating Characteristic (ROC) curves were utilized to ascertain the optimal cutoff values, which were subsequently converted into binary variables. A *p*-value of less than 0.05 was considered statistically significant.

## 3 Results

### 3.1 Information on post-CP complications

Among the enrolled patients, 35.1% (52/148) experienced postoperative complications. The cases included one instance of scalp infection, three cases of new-onset hydrocephalus, one instance of epilepsy, one instance of subcutaneous effusion with epilepsy, two instances of cerebral haemorrhage, one instance of cerebral haemorrhage with subdural haemorrhage, one instance of cerebral infarction, one instance of hydrocephalus with subdural effusion, six instances of subcutaneous effusion, one instance of subcutaneous effusion with epidural haematoma, one instance of subcutaneous effusion with subdural haematoma, two instances of subcutaneous effusion with both epidural and subdural haematoma, one instance of subcutaneous effusion with subdural haematoma, three instances of subcutaneous effusion with subdural effusion, nine instances of epidural haematoma with subdural haematoma, and one instance of epidural haematoma. As illustrated in Table 1, the following cases were observed: five cases of hematoma, two cases of epidural effusion, seven cases of subdural haematoma, and three cases of subdural effusion.

**Table 1:** Complications related to CP.

Complications	Number of complications	Percentage (%)
Extradural haemorrhage	16	30.8
Subdural haemorrhage	18	34.6
Hydrocephalus	4	7.7
Subdural effusion	7	13.5
Epilepsy	2	3.8
Epidural fluid	3	5.8
Intracranial infection	1	1.9
Scalp infection	1	1.9
cerebral hemorrhage	3	5.8
Subcutaneous effusion	14	26.9
Cerebral infarction	1	1.9

The total number of patients who developed complications after CP was 52. Patients may have multiple complications, so the sum of percentages exceeds 100%.

### 3.2 Univariate analysis of complications after CP

No statistically significant differences were observed between the postoperative complication group and the non-complication group with regard to age, gender, epilepsy, hypertension, diabetes, repair interval duration, skin flap shift, displacement direction, or cranial bone resection size. Statistically significant differences were observed in relation to the type of repair material used, the volume of blood loss, and the skin flap shift (Table 2).

**Table 2:** Univariate analysis of complications after CP.

Factors (self-variable name)	non-complication (*n* = 96)	postoperative complication (*n* = 52)	*χ^2^/Z/t*	*p*-value
Age [Years, median (IQR)]	52.5 (41.25, 59.00)	51.5 (43.00, 56.75)	−0.28	0.780
Gender [*n*, %]				
Male	70	40		
Female	26	12	1.868	0.172
Epilepsy [*n*, %]				
No	4	5		
Yes	92	47	1.219	0.270
Context for craniectomy [*n*, %]				
Trauma	61	34		
Stroke	35	18	0.050	0.823
High blood pressure [*n*, %]				
No	59	37		
Yes	37	15	1.390	0.238
Diabetes [*n*, %]				
No	89	48		
Yes	7	4	0.008	0.929
Intraoperative bleeding [ml, median (IQR)]	100 (100, 200)	200 (100, 300)	−2.081	0.037
CP material [*n*, %]				
Autologous bone	10	2		
Titanium mesh	65	29		
PEEK	10	2	6.625	0.036
Time to CP [month, median (IQR)]	4 (3, 6)	4 (2, 6)	−0.110	0.909
Craniectomy side [*n*, %]			0.947	0.344
Left	50	34		
Right	46	18	2.431	0.119
Axial Craniectomy Dimension [mm, Median (IQR)]	43.4 (40.4, 46.1)	41.5 (39.1, 44.2)	−0.81	0.423
Skin Flap Shift [mm, median (IQR)]	10.24 (5.49, 13.42)	13.68 (9.12, 19.02)	−3.46	<0.001
Skin flap shift direction [*n*, %]				
Positive	66	30		
Negative	30	22	1.81	0.179

### 3.3 Multifactorial analysis of post-CP complications

Multivariate analysis incorporated epilepsy, graft material, skin flap shift, displacement direction, and displacement distance. Continuous variables underwent ROC curve analysis to determine optimal cut-off values, yielding 13.55 mm for displacement distance and 175 mL for blood loss. Prior to the final analysis, the transformed variables were examined for multicollinearity. The results, presented in Table 3, indicated that multicollinearity was not a concern in the model. After conversion into categorical variables for multivariate logistic regression, and following confirmation of adequate model fit by a non-significant Hosmer-Lemeshow test (*p* = 0.941), skin flap shift was independently associated with postoperative complications following CP (Table 4).

**Table 3:** Multicollinearity diagnosis of candidate independent variables.

Factors(self-variable name)	VIF
Epilepsy	1.066
CP material	1.012
Craniectomy side	1.033
Skin flap shift direction	1.050
Skin flap shift	1.104
Intraoperative bleeding	1.060

**Table 4:** Multiple analysis of complications after CP.

Factors	*B* value	S.E. value	*p*-value	Exp (*B*)	Exp (*B*) 95% C.I.	
					Lower limit	Upper limit
Epilepsy	0.663	0.795	0.426	1.882	0.3966	8.944
CP material	−0.280	0.850	0.108	0.742	0.143	4.004
Craniectomy side	0.594	0.391	0.128	0.842	0.246	1.160
Skin flap shift direction	0.506	0.401	0.207	1.658	0.756	3.640
Intraoperative bleeding	0.594	0.391	0.128	0.842	0.842	3.899
Skin flap shift	1.175	0.410	0.004	3.239	1.450	7.237

### 3.4 Assessment of the predictive value of skin flap shift based on ROC curve for complications after CP

**Figure 3:**
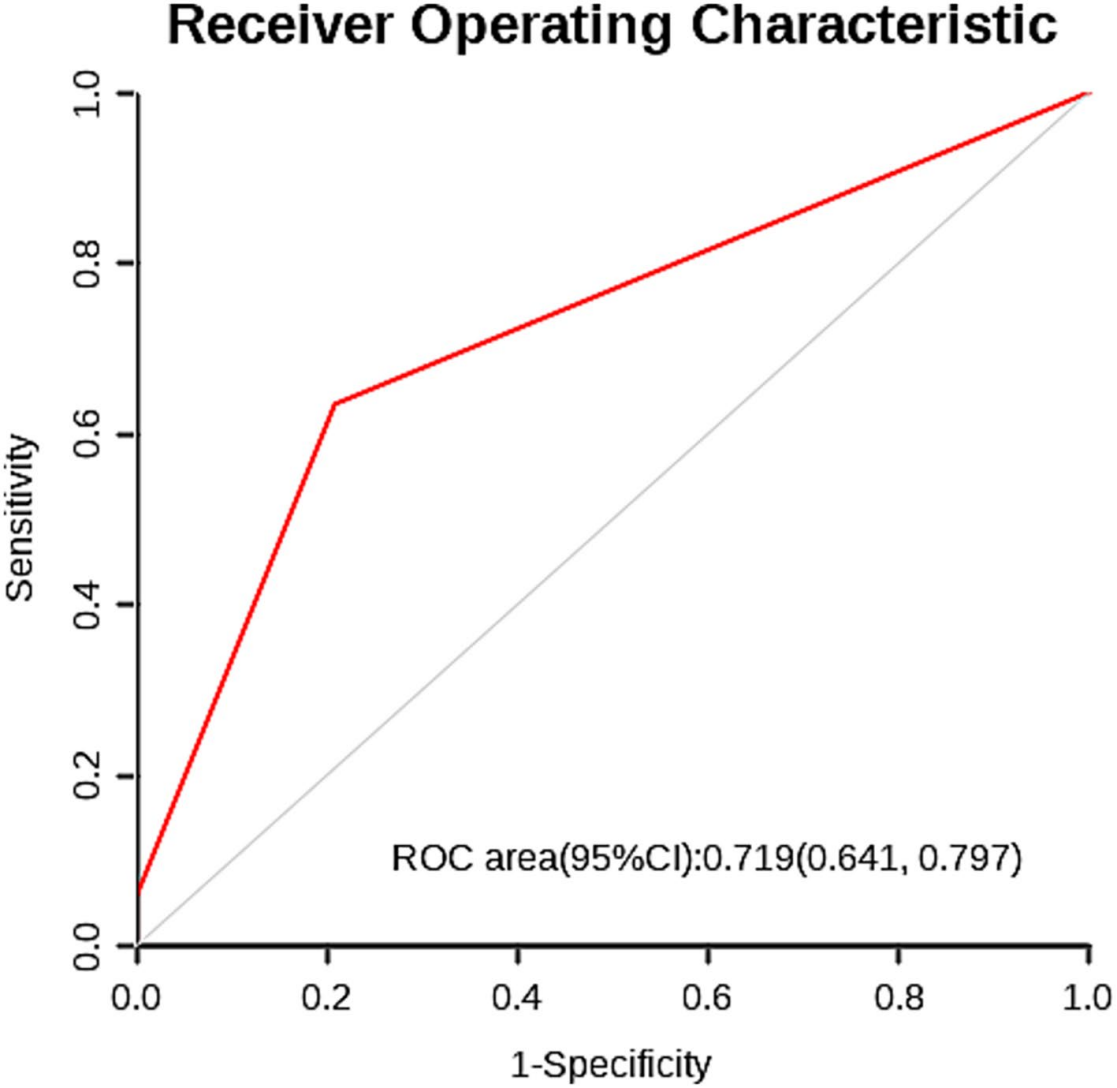
The ROC curve for predicting complications after CP based on flap displacement (cut-off value: 13.55 mm) shows an area under the curve of 0.719 (95% confidence interval: 0.641–0.797).

Based on the multi-factor analysis, skin flap shift was classified into two categories, using the occurrence of complications after CP as the predictive indicator, and analyzed using the ROC curve. It was found that the area under curve was 0.719 (95%CI: 0.646–0.797) (Figure 3).

## 4 Discussion

Cranial reconstruction not only prevents secondary brain tissue damage caused by cranial defects but also alleviates the psychological burden patients experience due to such defects (13). Although certain postoperative complications following cranioplasty may be managed through pharmacological or conservative treatment without severe consequences, some patients remain susceptible to adverse effects despite active intervention (14). Consequently, the precise preoperative prediction of complication risks and the development of personalized treatment plans for high-risk patients hold significant clinical importance.

Postoperative skin flap shift following CP is not merely a morphological alteration, but rather the core mechanical initiating factor triggering a series of complications (15). Its key hazards lie in not only potentially compromising the anatomical integrity of the surgical site, leading to localized blood supply insufficiency or even tissue necrosis, but also increasing the risk of infection, fluid accumulation, and neurological dysfunction. This study’s univariate analysis first established an association between flap displacement and postoperative complications following CP (16). Subsequently, a ROC curve determined the optimal cutoff value for skin flap shift. This value was then converted into a binary variable and incorporated into a multivariate logistic regression model, ultimately confirming that skin flap shift is independently associated with postoperative complications following CP.

These observations align with previous literature showing that reduced flap perfusion, excessive tension, and impaired venous drainage significantly increase infection risk and poor wound healing. Studies using laser-Doppler flowmetry and skin-perfusion imaging have demonstrated that even modest increases in flap tension can reduce microcirculatory flow by 20–40%, promoting tissue breakdown and fluid accumulation (17). Although such physiological data were unavailable in the present study, the displacement distance observed on preoperative CT may serve as a practical surrogate for tension-induced perfusion impairment.

The objective of ideal CP is to achieve precise alignment between the flap and bone window margins, thereby restoring cranial cavity integrity (18). However, any displacement of the flap, whether concave or convex, inevitably results in the creation of dead space. This area of dead space is susceptible to accumulation of blood, serous fluid, and inflammatory exudate (19). The consequence of this is twofold: firstly, it provides a favorable environment for bacterial proliferation, and secondly, it disrupts the normal fluid dynamics within the cranial cavity (19). It has been hypothesized that this may result in impaired cerebrospinal fluid circulation and absorption, which may consequently increase the risk of postoperative infection and subcutaneous fluid/haematoma accumulation (20).

Beyond the absolute displacement distance, the biomechanical behavior of the scalp flap may vary depending on the pattern of movement. In the domain of reconstructive surgery, the deformation of flaps can entail advancement, rotation, or transposition components. These variations in deformation result in distinct vectors of tension and vascular stretch (21). Although routine cranial CT cannot fully delineate these dynamics, certain radiologic features may provide indirect clues. Findings such as asymmetric thickening, contour bulging, or directional drift toward the defect can suggest altered biomechanical behavior. Intra-operatively, most displaced flaps demonstrated an advancement-dominant deformation pattern. Rotation or transposition type displacement was observed less frequently. These variations may influence factors such as perfusion, deep-tissue shear stress, and dead space formation. As a result, they may contribute to postoperative fluid accumulation or infection (22). Future imaging based biomechanical analyses are warranted to determine whether specific displacement patterns confer differential risk.

The initial hypothesis that the direction of skin flap shift would correlate with postoperative complications was not supported by the data. Specifically, in the univariate analysis, no significant association was found between the direction of shift and the incidence of complications (*χ*^2^ = 1.81, *p* = 0.179). Similarly, in the multivariate logistic regression analysis that adjusted for potential confounders related to patients, yielded a non-significant odds ratio for the direction of shift (OR = 1.65, 95% CI: 0.75–3.64, *p* = 0.207). These results indicate that, based on the current dataset, the direction of flap shift does not appear to be a significant predictor of postoperative complications. However, this lack of significance may be attributable to study limitations. Firstly, the limited sample size precluded further subtyping of postoperative CP complications, potentially masking direction-specific effects. Secondly, specific skin flap shift directions may correlate with particular subtypes, which could not be adequately explored. Thirdly, current definitions and measurement methods for skin flap shift direction require refinement to improve precision and reproducibility. It is recommended that future studies be conducted to explore the association between flap displacement direction and postoperative CP complications. Such studies would be improved by the expansion of sample sizes and subtyping of complications. In subsequent work, our team will undertake a more thorough validation of this hypothesis.

The present study incorporated two additional elements into the analysis. Firstly, the direction of skin flap shift was taken into consideration. Secondly, the absolute value of skin flap shift, measured by CT, was incorporated. The findings of the study indicated a substantial correlation with postoperative complications following CP: patients with skin flap shift distances exceeding 13.55 mm demonstrated a 3.239-fold higher incidence of postoperative complications compared to those with displacement distances ≤13.55 mm. From a clinical standpoint, these findings carry several important technical implications. A tension free scalp closure is essential to prevent vascular congestion and reduce postoperative fluid accumulation. Preserving the vascular pedicle during flap elevation is also critical, as excessive manipulation may impair perfusion. In addition, meticulous hemostasis before closure remains a key strategy in minimizing complications, particularly in patients with marked preoperative flap displacement. The use of three-dimensional navigation systems and patient specific implants may further enhance contour matching, reduce dead space, and decrease mechanical stress on the flap. Collectively, these approaches may help lower complication rates in patients with pronounced flap shift. Future large scale quantitative studies are needed to develop risk-stratification models for flap displacement across different brain regions. Such research will help generate more precise evidence and support the standardization of CP surgical procedures.

Furthermore, the study identified several additional variables that exhibited no statistically significant association with complications following cranioplasty. These significant negative findings necessitate further investigation. In univariate analysis, no significant differences were demonstrated between the complication group and the non-complication group with respect to age or pre-existing conditions. This finding is in contrast with the results of previous studies which suggested an increased surgical risk with advancing age, a phenomenon that may be attributable to age-related declines in tissue repair capacity and immune function (23–25). However, the median age of the cohort was 52 years, with a narrow interquartile range (42–58), indicating minimal variability in age-related risk factors that may have obscured potential associations.

There was no significant association with comorbidities or complications such as epilepsy, hypertension, or diabetes. This finding is somewhat unexpected, given the widely recognized impact of diabetes on wound healing and increased infection risk in surgical populations (26). Hypertension and epilepsy, on the other hand, are associated with broader cardiovascular and neurological vulnerability (27, 28). A potential explanation for this phenomenon may be found in the study’s inclusion criteria. Patients with systemic diseases that severely impair healing were excluded from the study, potentially homogenizing the comorbidity burden within the cohort and thereby diminishing the impact of these conditions. Furthermore, rigorous perioperative management may have mitigated these risks, underscoring the role of clinical interventions in counteracting the harms associated with comorbidities.

The present study is subject to the following limitations: Firstly, the sample size was relatively small, and the conclusions drawn require further validation in large-scale cohort studies. Secondly, given that all research data originated from a single center, there is a possibility that selection bias may have been introduced; the representativeness of the data requires assessment through multicenter prospective validation with standardized imaging-based measurements and stratified analyses across different displacement patterns. Thirdly, the present study was limited to the inclusion of short-term complications. As late bone flap resorption or delayed infection may manifest several months to years following cranioplasty (25), further long-term studies are warranted to explore the correlation between the uniqueness of skin flap shift and long-term complications.

## 5 Conclusion

Our retrospective analysis reveals an independent association between the distance of skin flap shift and postoperative complications after CP surgery, positioning it as a potential novel predictor. This observation implies a correlation that merits further investigation in preoperative assessments. Consequently, it is hypothesized that for patients with a skin flap shift greater than 13.55 mm, enhanced perioperative management might be considered, with the aim of potentially improving outcomes.

## Data Availability

The raw data supporting the conclusions of this article will be made available by the authors, without undue reservation.
